# 3DCONS-DB: A Database of Position-Specific Scoring Matrices in Protein Structures

**DOI:** 10.3390/molecules22122230

**Published:** 2017-12-15

**Authors:** Ruben Sanchez-Garcia, Carlos Oscar Sanchez Sorzano, Jose Maria Carazo, Joan Segura

**Affiliations:** GN7 of the Spanish National Institute for Bioinformatics (INB) and Biocomputing Unit, National Center of Biotechnology (CSIC)/Instruct Image Processing Center, 28049 Madrid, Spain; rsanchez@cnb.csic.es (R.S.-G.); coss@cnb.csic.es (C.O.S.S.)

**Keywords:** protein structure, protein databases, machine learning, position-specific scoring matrices

## Abstract

Many studies have used position-specific scoring matrices (PSSM) profiles to characterize residues in protein structures and to predict a broad range of protein features. Moreover, PSSM profiles of Protein Data Bank (PDB) entries have been recalculated in many works for different purposes. Although the computational cost of calculating a single PSSM profile is affordable, many statistical studies or machine learning-based methods used thousands of profiles to achieve their goals, thereby leading to a substantial increase of the computational cost. In this work we present a new database compiling PSSM profiles for the proteins of the PDB. Currently, the database contains 333,532 protein chain profiles involving 123,135 different PDB entries.

## 1. Introduction

Position-specific scoring matrices (PSSMs) have been used in many works to compute and predict a broad range of protein features. For example, PSSM profiles have been used to predict residue solvent accessibility [1], protein secondary structure [2], residue-residue contact maps [3], protein disordered regions [4], protein binding sites [5], protein-DNA interactions [6] or protein-protein interface hotspots [7]. Although these works used different prediction algorithms and methodologies, they share a common procedure that can be found in many other publications. This procedure can be summarized as follows. First, a particular protein feature is collected from structural models and annotated over their amino acid sequences. Second, PSSM profiles are computed and used to characterize protein amino acids. Finally, a machine learning algorithm fed with PSSM profiles is trained to predict the selected feature over protein sequences or structures.

Several resources compiling PSSM profiles are currently available. The Conserved Domain Database (CDD) [8] annotates the location of conserved domains in proteins by means of PSSM profiles. However, these PSSM profiles are not computed on the whole protein sequence, but over protein domains defined the by Pfam [9], SMART [10], COG [11] or TIGRFAM [12] databases. The MulPSSM [13] and 3PFDB database [14] also contain multiple PSSM profiles for protein domains according to the Pfam classification. Finally, the Gene3D [15] and SUPERFAMILY [16] databases annotate PSSM profiles on proteins and genomes using hidden Markov models (HMM) of the CATH [17] and SCOP [18] databases, respectively. Although these resources compile amino acids profiles, only those protein regions that fall within protein domains are annotated and, thus, no PSSM profiles are available for non-domain residues.

In this work we present 3DCONS-DB, a database of PSSM profiles computed over protein sequences collected from the Protein Data Bank (PDB) [19]. The main difference of 3DCONS-DB with respect to the databases described above is that, in 3DCONS-DB, the PSSM profiles have been computed over whole protein sequences and, thus, they cover domain and non-domain regions. Many protein features, such as binding sites, post-translational modifications (PTMs), short linear motifs (SLiMs) [20], or disordered regions, may occur in regions comprised outside domains, which suggest that non-domain regions are worthy of studying. To confirm the relevance of non-domain regions, we have compared the occurrence of several functional features in domain and non-domain residues. Our analysis shows that non-domain regions seem functionally relevant and that the amount of information encoded in their PSSM profiles is around 80% of the information encoded in domain regions. Moreover, 3DCONS-DB is a valuable resource to avoid the recalculation of PSSM profiles for PDB entries and, thus, facilitates the development and testing of prediction methods that use PSSM information. Currently, our database contains PSSM profiles for 333,532 protein chains involving 123,135 different PDB entries. Also, a web application is available to access 3DCONS-DB data, including a REST interface to access data programmatically, allowing for the compilation of PSSM profiles in a ZIP file and visualization of PSSM data through a web browser. The database is freely available at http://3dcons.cnb.csic.es.

## 2. Results

Currently, 3DCONS-DB database compiles PSSM profiles for 123,135 PDB entries, involving 333,532 protein chains and 83,297 non-redundant protein sequences. The database is freely available and accessible either through a browser or using a web service designed for programmatic access. In this section we also present the analysis and comparison of PSSM profiles in protein domain and non-domain regions and show how 3DCONS-DB data can be used to predict secondary structure and residue contact numbers.

### 2.1. Domain and Non-Domain Region Analysis

One of the aims of this work is to show that non-domains regions of proteins are functionally relevant and, therefore, having profiles characterizing non-domain residues (in addition to profiles of domain residues) will benefit the calculation and prediction of biological features in these regions. To that end, we have measured the occurrence of different biological features in protein domain and non-domain regions. Protein domains were determined in terms of the Pfam classification [9] for each protein of the PDB. After the analysis of all PDB chains, we found that the average size of protein chains are 253 residues, with 78% of residues falling within well-defined protein domains; therefore, most PDB chains are predominantly composed of domain regions and non-domain residues represent a minor fraction (22%). The question then arises: are non-domain residues functionally relevant? To answer this question, we have analyzed how often different biological features such as secondary structure, binding sites, PTMs, SLiMs and genomic variants associated to diseases occurred in domain and non-domain regions.

Table 1 shows the distribution of secondary structure elements, binding site residues, PTMs and genomic variants associated to diseases that were found in domain and non-domain regions for the proteins of the PDB. In terms of secondary structure, we found that 81% of all residues that dssp software classified in some secondary structure category fall in domain regions. Therefore, domain residues tend to form secondary structures more often than non-domain residues. Binding sites residues are equally distributed in domain and non-domain regions, thus, the proportion of binding sites in domain and non-domain regions is the same as the proportion of all residues; therefore, both type of regions seems to have a similar role driving protein interactions. In terms of PTMs, we found that 38% of residues affected by PTMs were located in non-domain regions and, therefore, more than the expected number if they were uniformly distributed (22%). A similar result was obtained for the analyzed SLiMs, with 37% of them occurring in non-domain regions. Finally, the distribution of genomic variants associated to diseases follows a similar distribution as all residues, so that domain and non-domain regions seems to be equally affected by mutations that cause diseases. In general, we can observe a uniform distribution of these features, except for PTMs and SLiMs that occurred more often in non-domain regions than expected. These results, which are in line with other studies [21,22], suggest that non-domain regions have an active role in protein signaling.

Another important comparison is the quality and amount of information of PSSM profiles in domain and non-domain residues. To estimate these values, we analyzed the multiple sequence alignments (MSAs) that can be obtained by stacking the aligned sequences computed by PSIBLAST. Table 2 shows the fraction of gaps and the mean entropy calculated in domain and non-domain positions.

For each PDB chain, PSIBLAST recovered an average number of 243 protein sequences that were used to generate a MSA. Pfam domains are computed from MSAs collected from non-redundant sets of protein sequences and, thus, the number of gaps is expected to be smaller in domain than non-domain positions. In our analysis we observed that the gap frequency was 1.8% in domain and 10.5% in non-domain positions, in agreement with the expected results. However, the gap frequency was 10 times greater in non-domain regions, we obtained an average number of 217 protein sequences that were aligned in these positions with no gaps. Then, to measure whether these aligned sequences contained more information than random alignments we computed the Williamson entropy [23] (see Section 4.1) for the MSAs. The entropy values for domain and non-domain positions (Table 2), as expected, are higher in domain than in non-domain regions. However, the entropy value of non-domain positions is around 80% of the entropy scores of domain sites and higher than the expected value of a random alignment (a random alignment would produce a Williamson entropy value of 0).

### 2.2. Secondary Structure Prediction with 3DCONS-DB

**Figure 1:**
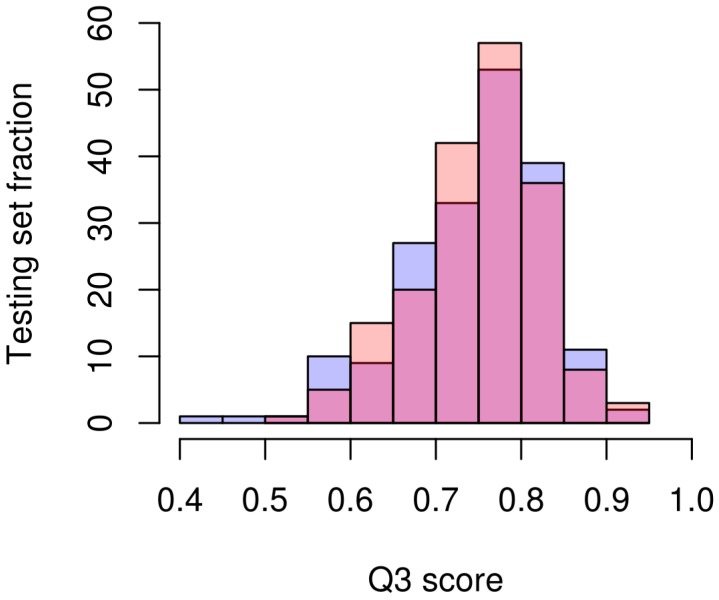
Q3 scores histogram. Histogram of Q3 scores predicting secondary structure in the testing set sequences. In blue color, obtained results using the original PSSM profiles. In pink color, obtained results using current 3DCONS-DB PSSM profiles.

As a concrete example of how 3DCONS-DB has a clear impact in the agile development of new bioinformatics tools, we used 3DCONS-DB data to train a neural network classifier for protein secondary structure prediction. The selected neural network architecture was the same as the one described in PSIPRED [24], consisting on two sequential neural networks of 75 and 65 hidden units, respectively, that where fed with PSSM profiles over a sequence window of 15 amino acids. For training and testing we used the same methodology and PDB entries proposed by Jones [24]. Calculating the new PSSM profiles for testing and training sets involved computing three iterations of PSIBLAST for 2245 protein sequences. Using a 32-core (i7 2.4 GHz) workstation, this step took over 178 h of computation, that is, more than one week. Figure 1 shows the Q3 performance (percentage of correct predictions in a three-class classification problem) of the predictions in the testing set using the original and 3DCONS-DB PSSM profiles. The Q3 average using original and 3DCONS-DB PSSM profiles was 74.6% and 75.1% with a standard deviation of 8.2% and 7.3%, respectively. Leaving aside Q3 improvement, the important result is that contrary to the 178 h used to compute the PSSM profiles, training and testing the network took only over 40 min using a laptop with a GPU NVIDIA GTX 960M. Therefore, having the PSSM profiles available for any PDB entries speed up the process for training and benchmarking for methods that use this type of data. Table S1 of Supplementary Material shows the computation time of calculating PSSM profiles compared to retrieving them from 3DCONS-DB.

### 2.3. Residue Contact Number Predition with 3DCONS-DB

As a second example of how 3DCONS-DB can simplify and facilitate the development of algorithms for the prediction of protein structural features, we have trained a support vector regression (SVR) model for predicting residue contact number (CN) using the same procedure as described in Yuan et al. [25]. In their work, Yuan et al. defined the CN of a residue as the number of C-beta atoms of other residues that are within a sphere of a given radius centered at its C-beta atom. The model was trained using PSSM profiles over a sliding window of 15 amino acids. To measure the computing time, we calculated the PSSM profiles for each of the 945 PDB chains proposed to train and test the method. This process took more than 48 h using our 32 cores (i7 2.4 GHz) workstation while training and testing the SVR was performed in less than 8 h using the same computer.

The evaluation consisted in a threefold cross-validation using different distance thresholds to define contacts between C-beta atoms. Table 3 shows the root mean square error (RMSE) of the normalized CN in the original work and using 3DCONS-DB data. In this example, the performance improved when 3DCONS-DB data was used; however, the important result is that while computing the PSSM profiles took more than 48 h, training and testing the model took less than 8 h.

## 3. Discussion

3DCONS-DB is a new database that compiles PSSM profiles for PDB protein sequences with the aim set at facilitating the development and testing of prediction methods that use PSSMs. Currently, the database contains 123,135 PDB entries, involving 333,532 protein chains and 83,297 non-redundant protein sequences. The main difference with similar resources is that 3DCONS-DB annotates residues over whole protein sequences and not only on domain regions. However, the comparison of different biological features on domain and non-domain residues indicates that both types of protein regions seem functionally relevant. Indeed, non-domain residues are most often affected by post-translational modifications that domain ones and short linear motifs can be found more frequently in them as well, indicating that non-domain regions might be more involved in protein signaling than domain regions.

## 4. Materials and Methods

### 4.1. Comparison of Domain and Non-Domain Regions

Several biological features to characterize and compare protein domain and non-domain regions were collected from different sources. Secondary structure was calculated using the dssp software [26] for all PDB entries. Binding sites residues were determined using a distance threshold of 8 Å between all PDB chain pairs. PTMs and other functional features were collected from PhosphoSitePlus [27] through 3DBIONOTES [28,29] web services. SLiM information was gathered form ELM (Eukaryotic Linear Motif) database [20]. ELM database compiles predicted and experimental information curated from the scientific literature. In this work, only manually curated SLiM were used to characterize domain and non-domain regions. Finally, genomic variants were retrieved from BioMuta database [30]. These features were mapped on PDB protein residues to analyze and compare the biological relevance of non-domain region, as compared to protein domains.

To measure the amount of information encoded behind PSSM profiles, we have calculated the Williamson entropy [23] values of the MSA positions that can be built from the PSIBLAST outputs. Williamson entropy measures the amount of information for a given position normalizing each frequency class by its global frequency in the MSA. For each position the entropy can be calculated using the expression:

(1) $$\overset{k}{\underset{i=1}{\sum }}p_{i}\ln\frac{p_{i}}{\bar{p_{i}}}$$

where $p_{i}$ is the frequency of the class i in the particular position and $\bar{p_{i}}$ is the global frequency of the class i in the MSA. We have used two different sets of classes: (1) the originally proposed set of classes, k=9 where amino acids are grouped into categories depending on their physicochemical features: VLIM, FWY, ST, NQ, HKR, DE, AG, P and C; (2) the 20 naturally occurring amino acids as the set of classes (*k* = 20) in order to ensure that the computed entropy value was not magnified due the class amino acid reduction.

### 4.2. Database and Web Server

3DCONS-DB data was compiled computing the iterative BLAST algorithm (PSIBLAST) [31] with default parameters on protein sequences collected from the PDB. We computed three iterations of PSIBLAST for each individual chain of the different PDB entries using the non-redundant protein sequence database UniRef100 [32] as reference. Currently, 3DCONS-DB contains PSSM profiles for 123,135 PDB entries and 333,532 protein chains involving 83,297 non-redundant protein sequences. Compiling this information took over 1650 h using 128 cores (i7 2.4 GHz). The results were stored in a SQL database (https://www.sqlite.org) and a web server was built to dispatch the data. The web server was developed using the Ruby on Rails framework (http://rubyonrails.org) and was designed to collect and deliver PSSM profiles of PDB entries. 3DCONS-DB data can be accessed in three different ways: through a REST web service to retrieve PSSM profiles in JSON format, submitting a list of desired PDB ids and retrieving their PSSM scores in a ZIP file and, finally, using 3DCONS-DB web application to explore specific PSSM profiles through a browser.

### 4.3. The Web Client

The web client was designed to display 3DCONS-DB data on a browser and to provide an interactive environment to explore PSSM profiles at sequential and structural level. The information is divided in three major panels (see Figure S2 of Supplementary Material, Section S1): the structural viewer, the global PSSM profile and the residue level PSSM table. The structural panel integrates the NGL 3D viewer [33] to display protein structures and to represent PSSM scores over them. The global PSSM profile panel was built using the D3 JavaScript library (http://d3js.org) and it summarizes PSSM scores for the entire selected sequence. Finally, the residue level PSSM table contains the exhaustive PSSM score list for each residue of the selected protein. 3DCONS-DB client can display the different levels of PSIBLAST information; thus, the scores of the different iterations, swapping between PSSM scores and position-specific frequency matrix scores, or exploring the PSSM scores for the different chains of a PDB entry.

## Figures and Tables

**Table 1:** Biological features of domain and non-domain residues for Protein Data Bank (PDB) proteins.

	Residues (%) ^1^	SS (%) ^2^	BS (%) ^3^	PTM (%) ^4^	SLiM (%) ^5^	Variants (%) ^6^
Domain	78	81	78	62	63	77
Non-domain	22	19	22	38	37	23

^1^ Percentage of residues in domain and non-domain regions; ^2^ Percentage of secondary structure elements in domain and non-domain regions; ^3^ Percentage of binding site residues in domain and non-domain regions; ^4^ Percentage of posttranslational modifications in domain and non-domain regions; ^5^ Percentage of short linear motifs in domain and non-domain regions; ^6^ Percentage of genomic variants associated to diseases. Post-translational modifications (PTMs); short linear motifs (SLiMs).

**Table 2:** Information per position of position-specific scoring matrices (PSSM) profiles in domain and non-domain regions.

Region ^1^	Gap Freq. (%) ^2^	Entropy ^3^	Entropy ^4^
Domain	1.8	1.36	1.97
Non-domain	10.5	1.11	1.62

^1^ Location; ^2^ Gap frequency in the MSA; ^3^ Williamson entropy grouping the amino acids in nine classes; ^4^ Williamson entropy using the 20 naturally occurring amino acids.

**Table 3:** Root mean square error predicting residue contact number.

Threshold ^1^	8 Å	10 Å	12 Å	14 Å
Yuan et al. ^2^	0.77	0.75	0.72	0.72
3DCONS-DB ^3^	0.62	0.64	0.68	0.69

^1^ Distance threshold used to define contact between C-beta atoms; ^2^ Root mean square error reported in Yuan et al. work [25]; ^3^ Root mean square error using 3DCONS-DB data to train and test the support vector regression model.

## Supplementary Materials

The following are available online, Figure S1: 3DCONS graphic interface, Table S1: Computation time of profiles.
