# Supplementary material for: 3DCONS-DB: A Database of Position-Specific Scoring Matrices in Protein Structures
Source: Molecules. 2017 Dec 15;22(12):2230. doi: 10.3390/molecules22122230 (PMC6149929; doi:10.3390/molecules22122230)
Supplement: Supplementary file 1 [file molecules-22-02230-s001.pdf]

3DCONS-DB: A database of structural position specific scoring matrices

Ruben Sanchez-Garcia, C.O.S. Sorzano, J.M. Carazo and Joan Segura

S1 PSSM computational cost analysis

Table S1 shows the computation time required to calculate PSSM profiles compared to retrieving them from 3DCON-DB. For this analysis, the PSSM profiles were computed using 32 cores (i7-2.4Hz) and 1Gb of bandwidth.

Number of sequences[1]	Computation time[2] (h)	3DCONS-DB time[3] (s)
100	6	<10
200	12	<10
500	30	<20
1000	61	<30
5000	303	<60
10000	605	<120

Table S1 PSSM profiles computation time. [1]Number of sequences for which the PSSM profiles were computed. [2]Computation time to calculate the PSSM profiles. [3]Time to retrieve the PSSM profiles from 3DCONS-DB.

S2 3DCONS-DB web client graphical user interface

3DCONS-DB provides a web application to explore PSSM profiles for individual PDB entries. The web application can be accessed at http://3dcons.cnb.csic.es/query_form where a web form is provided to query the database using a PDB code. The information is divided in three different panels (Figure S1). The structural panel (Figure S1A) displays the structure of the selected PDB, on the top of the panel a select menu can be used to switch which PSSM profile is displayed among the different chains. The global PSSM profile panel (Figure S1B) summarizes the PSSM scores for the whole sequence, displaying the amount of information for each amino acid position of the selected chain and also, the pfam domains that the sequence comprise. Finally, the PSSM profile panel (Figure S1C) displays all the PSSM scores for each residue of the selected chain. Last two columns are relative weight of gapless real matches to pseudocounts and information per position values as described in [1,2]. Structure and PSSM panel are interactively connected in such a way that clicking a row in the PSSM table will highlight the corresponding residue in the protein structure and clicking (shift+click) a reside in the protein structure will highlight the PSSM table row.

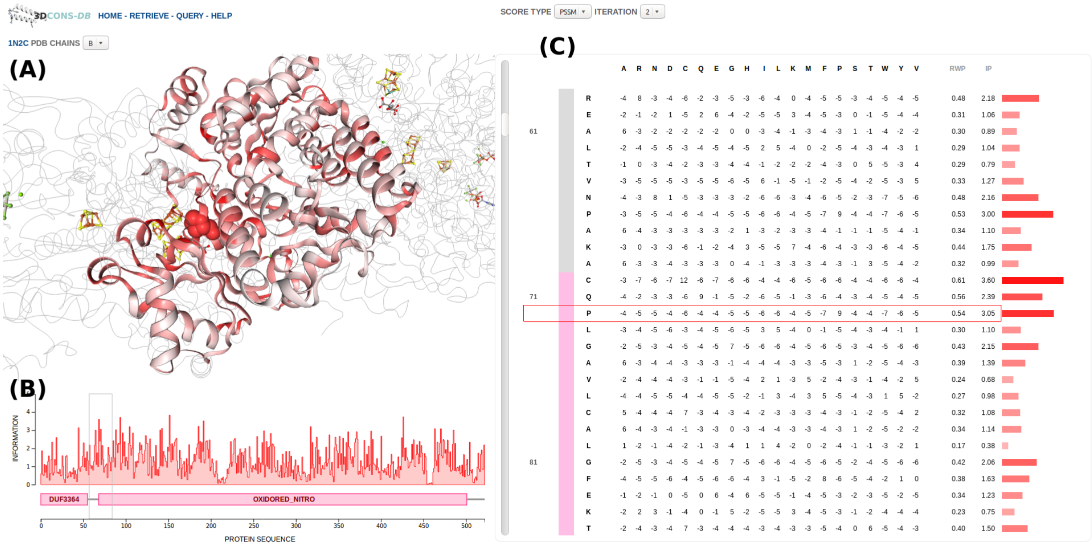

Figure S1 3DCONS-DB web client GUI. Screenshot of the 3DCONS web application corresponding to the case when information of the PDB entry 1N2C is being requested. (A) 3D structure of the PDB entry 1N2C. (B) Global PSSM profiles displaying the PSIBLAST amount of information per sequence amino acid position and Pfam domains. (C) PSSM profile panel, a table displays the exhaustive list of PSSM score for each residue.

REFERENCES

1. Altschul, S.F.; Madden, T.L.; Schaffer, A.A.; Zhang, J.; Zhang, Z.; Miller, W.; Lipman, D.J. Gapped blast and psi-blast: A new generation of protein database search programs. *Nucleic acids research* **1997**, *25*, 3389-3402.
2. Henikoff, S.; Henikoff, J.G. Position-based sequence weights. *Journal of molecular biology* **1994**, *243*, 574-578.
